# Patient-Derived Stem Cells, Another *in vitro* Model, or the Missing Link Toward Novel Therapies for Autism Spectrum Disorders?

**DOI:** 10.3389/fped.2019.00225

**Published:** 2019-06-06

**Authors:** Gilles Maussion, Cecilia Rocha, Geneviève Bernard, Lenore K. Beitel, Thomas M. Durcan

**Affiliations:** ^1^Department of Neurology and Neurosurgery, Montreal Neurological Institute and Hospital, McGill University, Montreal, QC, Canada; ^2^Departments of Neurology and Neurosurgery, Pediatrics and Human Genetics, McGill University, Montreal, QC, Canada; ^3^Division of Medical Genetics, Department of Internal Medicine, McGill University Health Center, Montreal, QC, Canada; ^4^Child Health and Human Development Program, Research Institute of the McGill University Health Center, Montreal, QC, Canada; ^5^MyeliNeuroGene Laboratory, Research Institute of the McGill University Health Center, Montreal, QC, Canada

**Keywords:** autism spectrum disorder, genetics, post-mortem brain studies, human induced pluripotent stem cells, therapeutic strategy

## Abstract

Autism Spectrum Disorders (ASDs) is a multigenic and multifactorial neurodevelopmental group of disorders diagnosed in early childhood, leading to deficits in social interaction, verbal and non-verbal communication and characterized by restricted and repetitive behaviors and interests. To date, genetic, descriptive and mechanistic aspects of the ASDs have been investigated using mouse models and post-mortem brain tissue. More recently, the technology to generate stem cells from patients' samples has brought a new avenue for modeling ASD through 2D and 3D neuronal models that are derived from a patient's own cells, with the goal of building new therapeutic strategies for treating ASDs. This review analyses how studies performed on mouse models and human samples can complement each other, advancing our current knowledge into the pathophysiology of the ASDs. Regardless of the genetic and phenotypic heterogeneities of ASDs, convergent information regarding the molecular and cellular mechanisms involved in these disorders can be extracted from these models. Thus, considering the complexities of these disorders, patient-derived models have immense potential to elucidate molecular deregulations that contributed to the different autistic phenotypes. Through these direct investigations with the human *in vitro* models, they offer the potential for opening new therapeutic avenues that can be translated into the clinic.

## Introduction: Genetics of Autism

Autism is a complex, multigenic and multifactorial neurodevelopmental disorder that was originally defined based on three clinical criteria (i) abnormalities in social interactions, (ii) deficits in verbal and non-verbal communication and (iii) the presence of stereotypical and repetitive behaviors (1). As the genetic causes of autism have been investigated, the literature now refers more to Autism Spectrum Disorders (ASDs) (2) rather than autism, originally defined by Kanner. ASDs can be classified in two main categories: non-syndromic and syndromic based on a lack of or association to clinical manifestations outside of the autistic features, respectively. The non-syndromic ASDs are diagnosed based on structured interviews (3, 4) during childhood, according to specific clinical criteria. They are divided between those caused by specific gene mutations (5), deletions, disruptions (6, 7) or copy number variations (8) leading to abnormal gene dosage. Idiopathic forms of autism, whose genetic bases remain unclear represent the majority of ASDs' cases. Syndromic forms of autism such as Fragile X (9) or Rett syndromes (10) along with Tuberous sclerosis (11) and others (see Table 1) are characterized by a specific set of clinical features as well as the autistic triad. They are often monogenic, genetically well characterized and account for 10–20% of ASDs' cases (18). Examples of specific set of clinical features that can be seen in the syndromic ASDs include facial dysmorphisms, epilepsy, intellectual disability, and systemic manifestations. These features are not observed in non-syndromic forms of autism (2).

**Table 1:** Syndromic forms of autism that have been investigated in iPSC-derived studies.

**Disease**	**Gene**	**Location**	**Function**	**Symptoms**	**References**
Fragile X Syndrome	*FMR1* (CGG expansion in the 5'UTR sequence)	Xq27.3	RNA-binding molecule/ Regulates local translation	Intellectual Disability, Developmental Delay, Social Impairment, Hyperactivity Machroorchidism	(9)
Rett Syndrome	*MECP2*	Xq28	Methyl CpG DNA binding molecule/Regulates gene transcription	Affects essentially Females (X-linked forms) Intellectual Disability, Stereotyped Behavior, Epilepsy, Regression during childhood	(10)
	*CDKL5*	Xp22.13	Serine/Threonine Kinase		(12)
	*NTNG1*	1p13.3	Presynaptic Ligand involved in Axonal projection		(13)
	*MEF2C*	5q14.3	MADS box transcription enhancer factor 2; DNA binding molecule		(14)
Tuberous Sclerosis	*TSC1*	9q34.13	Interact with the tuberin to negatively regulate the mTOR pathway	Multisystemic disease involving the brain (Learning Deficits, Intellectual Disability, Epilepsy, Structural Brain abnormalities), skin (e.g., hypomelanotic macules, shagreen patches, angiofibromas), eyes (e.g., angiofibromas), Kidneys, heart e.g., rhabdomyomas) and lungs (lymphangioleiomyomatosis)	(11, 15)
	*TSC2*	16p13.3	GTPAse activating protein/interact with the tuberin to regulate the mTOR pathway		
Phelan-McDermid Syndrome	*SHANK3*	22q13.33	Scaffold protein of the post-synaptic compartment. Involved in synapse formation and in dendritic spine maturation	Global Developmental delay, Hypotonia, Absence of Speech or Speech delay, Dysmorphic Features	(16)
FoxG1 Deletion Syndrome	*FOXG1*	14q12	Forked-head transcription factor; Involved in brain development	Microcephaly, partial or complete agenesis of the corpus callosum, Intellectual Disability, Epilepsy, Autistic Features	(17)

Studies on syndromic forms of autism, as well as candidate gene approaches and genome-wide association studies, have led to the identification of more than 70 loci for ASDs in the human genome (18). Those loci are categorized into specific gene functions such as developmental programs (19), transcriptional (20), and translational regulation (21), cell signaling (22), gene imprinting (23), dendritic trafficking (24–26), and activity-dependent brain development and neurotransmission (27). Complementing these genetic studies, work on post-mortem brain tissue and mouse models have helped us better observe and understand changes that may occur in the context of ASDs at the cellular and molecular levels.

Over the past decade, technology has developed to derive induced pluripotent stem cells (iPSCs) from the somatic cells of patients with many different types of diseases. In the context of ASDs, this approach enables us to differentiate and grow human neurons from patients, within a dish, presenting a new *in vitro* tool to decipher how perturbations in specific genes or pathways may be either involved or the causative mechanisms in the development of ASDs, with the ultimate goal of developing new therapeutics. Multiple genetic susceptibility loci identified in ASDs strongly implies that these disorders are linked to genetic variants and risk factors across multiple genes that are best studied in human models. This review aims to discuss the strengths and limitations of mouse models, human post-mortem brain tissue and iPSCs in studying ASDs, and to summarize how these studies combined have contributed to advance our understanding into the molecular and cellular mechanisms that potentially cause ASDs.

## Mouse Models in Autism Spectrum Disorders

Animal models have been a useful tool in understanding how genes linked to ASDs may contribute to the pathogenesis of these disorders. In order to study ASDs, a combination of genetic, chemically induced, and environmental models have been generated. In the genetic models, ASDs-associated genes have been inactivated to observe distinct phenotypes within the mice that can include altered gene expression, cell morphology, and behavioral or social deficits.

Syndromic form of autism that are genetically characterized such as Fragile X (FXS), Rett Syndrome, and Tuberous Sclerosis (Table 1) are studied in mouse models. At an adult stage, mice inactivated for *Fmr1* (coding for the RNA-binding protein FMRP which acts as a translational repressor), present abnormal dendritic spine morphology, although one limitation of this model is that autistic traits often differ from one strain to another (28). Restoration of the abnormal protein synthesis in *Fmr1* knockout mice by S6K1 (a translation regulator) deletion can stabilize neurological function (29). *Fmr1* knockout leads to a disruption of synaptic protein interactions that notably involve metabotropic glutamate receptor subunit 5 (mGluR5) and Homer scaffold protein. Homer1a acts as a dominant negative isoform that prevents the normal interaction with mGluR5, and its deletion rescues, in H1a/Fmr1 double knockouts mice, mGluR5 signaling (30).

Further studies highlighted the importance of synaptic transmission in FXS, connecting it also to impairment of the GABAergic system (31). Interestingly, the GABA receptor agonist THIP (gaboxadol) was shown to restore neuronal excitability in the *Fmr1* knockout mice (32). A cytoplasmic polyadenylation element-binding protein (CPEB) also binds to mRNA controlling neuronal translation and modulating synaptic function. *Frm1/Cpeb1* double knockout mice display an amelioration in morphological, electrophysiological and behavioral phenotypes associated with FXS showing the importance of translational homeostasis for neural function (33). Studies on animal models suggest that *Fmr1* inactivation leads to translational deregulations which underlie abnormalities in excitatory and inhibitory neurotransmission.

Rett Syndrome (RTT) is caused, in the majority of cases, by loss of function mutations in the *MECP2* gene (Table 1). Mouse models have helped to investigate the impact of *Mecp2* mutations in RTT pathogenesis. Mecp2 was shown to be critical for GABAergic neuronal function in Rett Syndrome (RTT) as *Viaat-Mecp2*^−/*y*^ mice lack Mecp2 in GABAergic neurons, causing RTT-like features that include the development of stereotypes, self-injury, compulsive behavior and progressive motor dysfunction (34). Activation of Mecp2 expression in knockout mice reverses neurological symptoms (35). Interestingly, a study with 7 weeks and 21 weeks female *Mecp2*^+/−^ mice showed behavioral and motor deficits that were identified in distinct genetic backgrounds However, some phenotypes were also identified in only one genetic background (36). This study stresses the importance of age and strain background selection for RTT social behavior research using *Mecp2*^+/−^ female mice.

Mutations in *TSC1* and *TSC2* are known to cause Tuberous Sclerosis (TSC) (Table 1). Actually, a functional interaction between those two signaling proteins is required for the activation of mTOR complex 2 (37). The mTOR pathway is an important regulator of mitophagy and autophagy (38), and its activation by IGF-1 or other small molecules, can promote reversion of the developmental alterations observed in TSC. Behavioral abnormalities in *Tsc1*^+/−^ and *Tsc2*^+/−^ are rescued by inhibition of mTORC1 with rapamycin treatment (39). A TSC mouse model with Tsc2 loss under a Purkinje cell promotor showed increased repetitive behavior in *Tsc2*f/-; Cre mice. These social behavioral deficits were also prevented with rapamycin treatment (40). These mouse models have shown that dysregulation of the mTOR signaling pathways, induced by *Tsc1* and *Tsc2* inactivation, results in a Tuberous Sclerosis-like phenotype.

Shank3 is a synaptic scaffolding protein and gene mutations have been associated with an ASD phenotype (41). Male heterozygous mice with deleted *Shank3* display impairments in NMDA receptor signaling function, synaptic trafficking (42), hippocampal excitatory transmission, and motor learning although no social interaction deficit is observed (43). Another study reported that Histone deacetylase 2 (HDAC2) is upregulated in *Shank3-deficient* mice and HDAC2 knockdown in the prefrontal cortex rescues social deficits (44). Treatment with romidepsin, a histone deacetylase (HDAC) inhibitor, increases NMDAR transcription levels, restores NMDAR synaptic function, and alleviated social deficits in Shank3-deficient mouse (44). This model shows that mutations in the gene coding for a synaptic protein leads to autistic-like behavioral phenotypes in the mouse.

Taken together these models can reveal the individual mechanisms involved in syndromic forms of ASDs serving as a platform for proof-of-principle studies. Although knockout mice can model monogenic genetically-defined ASDs, idiopathic forms of autism, likely caused by multigenic risk factors, cannot be investigated in animal models. Furthermore, regulatory and coding regions from mouse and human genomes have acquired a more complex epigenetic organization or significant differences in the coding mRNAs having been submitted to evolutionary selective pressures. Most importantly, the diagnosis of ASDs still relies on assessing behavioral phenotypes that remain difficult to mimic in mouse models. Thus, studies in human samples are crucial for a better understanding of ASDs.

## Post-Mortem Brain Studies in ASDs

Although animal models have partially recapitulated some of the behaviors observed in patients and provided insights concerning gene expression and morphological deregulation potentially relevant to the ASDs, none of these systems are entirely modeling the complexity of ASDs. Studies on post-mortem brain tissue from patients with ASDs have enabled researchers to observe changes at the cellular and molecular levels that could be implicated as causative factors in the disease's manifestations.

The brain regions of interest for these post-mortem studies were chosen based on (i) their function, (ii) their potential implication in the disease and (iii) on imaging studies. As such, several morphological studies have been performed on the cerebellum, which controls balance, posture, and regulation of fine movements (45) as it is thought to be involved in the repetitive behaviors associated with ASDs (46). The frontal cortex has also received much attention, given its role in executive function (47), decision making (48) and working memory and attentional processes (49). Furthermore, the frontal cortex is interconnected with the limbic system (including the amygdala and hippocampus), important for learning and memory through the superior temporal sulcus, and forms a network involved in social perception and cognition (50).

Morphological studies of the cerebellum have shown it to be decreased in size accompanied by a decrease in the overall number of Purkinje cells in autistic patients relative to controls (51–53). Similarly, the structure of frontal cortex had been examined and decreased size of the minicolumns was observed in patients with idiopathic ASDs (54, 55). Interestingly, studies have reported in patients with ASDs, (i) increase of cortical thickness (56), (ii) increase of head circumference (57) as well as, more specifically in the prefrontal cortex, an increase in the number of neurons (58). These findings suggest that changes in cell number and synapses in brains from autistic patients, modify the cortical organization, connectivity and network efficiency (59, 60).

Other morphological studies were conducted on post-mortem brain tissue from patients diagnosed with syndromic ASDs, namely Fragile X and Rett Syndromes, two disorders that present with opposing morphological phenotypes. A decreased number of dendritic spines has been observed in the frontal cortex and in the CA1 regions of patient with Rett syndrome (25, 61) whereas, an increased number of elongated and immature dendritic spines was observed in brain sections from Fragile X patients (62). Studies performed on human post-mortem brain tissue have shown abnormalities in dendritic arborization that are specific for syndromic autisms (Supplementary Table 1).

Besides these morphological studies, genes expression have also been assessed in brain areas from patients diagnosed with ASDs, either based on candidate or whole genome approaches. From one of these studies, the expression of the *RELN* gene, which had been associated with autism (63) was observed to be decreased in the cerebellum and in the cortex of patients with ASDs (64). Interestingly, increased methylation of the *RELN* promoter was also observed in the frontal cortex and cerebellum of patients with ASDs. Furthermore, concomitantly with decreased number and size of the Purkinje cells, that are GABAergic neurons, decreased expression of *GAD1* (65) and *Parvalbumin* mRNA (66), were observed in the frontal cortex and in the cerebellum, respectively, of ASDs patients. Based on a candidate gene approach, the expression of two autism-associated genes, *SLC25A12* and *MARK1* (24, 67), and *BDNF* (68, 69) were found to be increased in Brodmann Area 46 of patients with idiopathic ASDs autistic patients (24, 70, 71).

Whereas changes in the expression of genes associated with ASDs had suggested that deregulation in molecular mechanisms involved in the dendritic trafficking and the synaptic plasticity occurs in brain regions from ASD patients, high throughout analyses aimed at generating non-biased profiles of the whole transcriptome have also been conducted on cortical or cerebellar regions (72–75) Interestingly, these independent research projects have identified in brain regions of ASD patients, deregulations of biological processes that were expected regarding the functions of ASDs-associated genes whose expression changes had already been observed (see Table 2). The first study focused on the whole transcriptome in the temporal cortex of patients with idiopathic ASDs and provided evidence of increased expression of genes involved in the immune system as well as deregulation of genes involved in cell-cell communication and cell cycle (72). Two other studies which investigated expression profiles of coding genes in the BA9, BA41/42, and cerebellar vermis (73) or the non-coding transcriptome in frontal and temporal lobes (75) have shown, in the brain of patients with idiopathic ASDs, increased expression of genes involved in inflammatory processes as well as a decrease in the expression levels of neuronal genes including several involved in synapse functioning. Interestingly, Parikshak et al. have also observed deregulation of primate-specific long non-coding RNAs associated with autism (75). A fourth study revealed age-dependent differential expression profiles between controls and patients with idiopathic ASDs (74). In young patients, deregulated genes were involved in cell number, cortical patterning, and differentiation whereas deregulation observed in the older patients involved genes in signaling and repair pathways. Interestingly, a gene set enrichment analysis of the four studies mentioned above has provided evidence for a correlation between a decrease in expression of synaptic and mitochondrial genes within distinct brain regions from ASDs patients (86).

**Table 2:** Candidate genes of ASDs whose expression has either been assessed on post-mortem brain tissue and or in iPSC-derived cells.

**Gene**	**Name**	**Location**	**Function**	**Link to autism**	**References**
*RELN*	Reelin	7q22.1	Part of the extracellular matrix and has a crucial role in cell positioning and migration processes	Genetic Association, Trend toward increased methylation in promoter (Cerebellum)	(63, 65, 76)
*BDNF*	Brain Derived Neurotrophic Factor	11p14.1	Neurotrophic factor, promote cell survival, Involved in synaptic plasticity	Increased levels in blood samples from patients with ASDs, Increased expression in BA46	(68, 69, 71)
*MARK1*	Microtubule Affinity Regulating Kinase 1	1q41	Regulates the affinity between MAPs and microtubules	Genetic Association, Increased expression in BA46	(24)
*SLC25A12*	Solute Carrier family 25 member 12	2q31.1	Mitochondrial Aspartate Glutamate transporter	Genetic Association	(67, 70)
*GAD1*	Glutamate Decarboxylase	2q31.1	Catalyzes the production of GABA from L-glutamic acid	Decreased level in ASDs	(65)
*GRM5*	Glutamate Metabotropic Receptor 5	11q14.2-q14.3	Involved in the regulation of neural network activity and synaptic plasticity.	Increase expression in Fragile X Syndrome	(77, 78)
*TRPC6*	Transient Receptor Potential cation Channel subfamily C member 6	11q22.1	Receptor-activated calcium channel in the cell membrane. Activated by DiacylGlycerol	Disrupted in Cases with ASDs	(79)
*MBD5*	Methyl-CpG Binding Domain Protein 5	2q23.1	Methyl-CpG-binding protein	Microdeletion in cases with Autism	(80, 81)
*SATB2*	SATB homeobox 2	2q33.1	DNA Binding Protein	Disruption by Chromosomal Rearrangement in patient with ASDs	(7, 81)
*EHMT1*	Euchromatic Histone lysine Methyltransferase 1	9q34.3	Histone Methyl Transferase		(7, 82)
*TCF4*	Transcription Factor 4	18q21.2	Basic helix-loop-helix transcription factor		(7, 82)
*CHD8*	Chromodomain Helicase DNA binding protein 8	14q11.2	Chromatin remodeling protein		(6, 83, 84)
*GRIN2B*	Glutamate ionotropic Receptor NMDA type subunit 2B	12p13.1	Subunit of the NMDA receptor		(6, 7, 83, 85)

**Figure 1:**
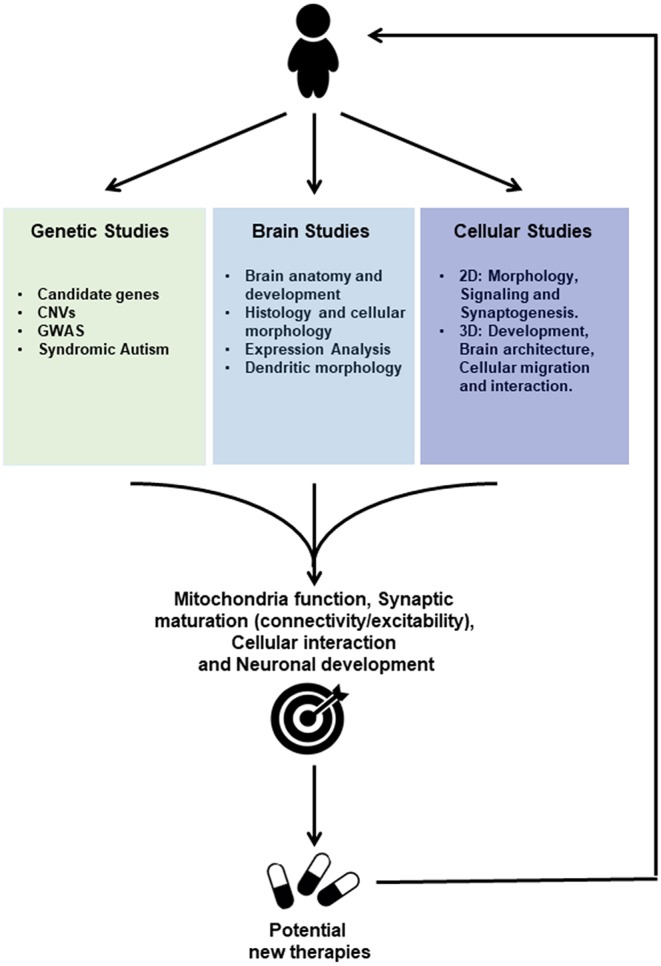
Scheme of ASD Drug discovery based on Genetic Studies, Post-mortem Brain Studies and Cellular Studies using induced pluripotent stem cells (IPSC) for future personalized treatment. Genetic studies reveal associations to Autism Spectrum Disorders either through candidate genes approach or Genome-wide Association Studies (GWAS), whereas genes mutated in syndromic autism have been identified. Furthermore, copy number variations (CNVs) that potentially lead to either gene disruption or duplication were more recently identified. Studies performed on post-mortem tissue provide information about brain anatomy and development, histology and cellular morphology, besides gene and protein expression profiles. Cellular Studies based on IPSC technology may be employed to generate neurons and other cell types (2D) and brain organoids (3D) using cells from patients. Those IPSC-derived models where used to investigate gene expression profiles and cellular morphology as well as cell signaling, synaptogenesis and electrophysiological properties. Moreover, 3D Studies allow to further investigate cellular migration and interaction during development. Those studies provide convergent information regarding the pathophysiology of the Autism Spectrum Disorders, toward altered mechanisms related to Mitochondria function, Synaptic maturation, Cellular interactions, and neuronal development. IPSC-derived cells are currently used to further investigate those altered functions and constitute human *in vitro* systems allowing drug screenings for potential targets that could lead to new therapies for ASDs.

These findings imply, that despite genetic and phenotypic heterogeneities, ASDs could be underpinned by deregulations of common molecular pathways and functions such as cell cycle, differentiation, mitochondrial function as well as synaptic plasticity and inflammation between brain regions; functions that had already been pointed out by genetics studies (Figure 1; Supplementary Table 1).

## Induced Pluripotent Stem Cells, Rescue, and Therapeutic Strategies

In the past decade, the technology has advanced to enable reprogramming of somatic cells from peripheral blood cells or skin fibroblasts into iPSCs (87) and *in vitro* generation of 2D or 3D neuronal culture from patients cells. While neurons grown in a dish enable researchers to investigate cell type-specific expression profiles and analyse electrophysiological properties of patient and control neurons, 3D organoids create a microenvironment that promotes interactions between cell types, leading to a more complete neuronal maturation (88) that is capable of recapitulating the organization of cortical layers observed during the neurogenesis (89).

Human iPSC-derived cells from patients with syndromic forms of autism such as Fragile X, Rett syndrome and tuberous sclerosis have been investigated and partially recapitulate phenotypes observed in post-mortem brain tissue or with pathological studies of rodent models of the diseases. Due to a lack of translational repression of mGluR5 mRNA at the synapse, iPSC-derived neurons from patients with Fragile X have an increased response to a group I mGluR agonist combined with a preferential differentiation into glutamatergic neurons (78); treatment with a mGLUR5 antagonist reduces the activation of the receptor and lead the Fragile X progenitors cells toward glial differentiation. A second study showed an increased response to Ca^2+^ permeable AMPA and NMDA receptors in iPSC-derived neurons from Fragile X patients (90), while treatment with a GluA2-lacking/calcium permeable-AMPARs antagonist reduces the length of the dendrites.

IPSC-derived Purkinje cells from patients diagnosed with tuberous sclerosis present with an increasing proportion of KI67 positive cells, decreased FMRP expression and an increased number of neurites (91). Human iPSC-derived neurons from patients with *TSC2*^+/−^ mutations were defective in mitophagy with (i) an accumulation of mitochondria in the dendrites and (ii) a reduced mitochondrial potential membrane (92). These findings corroborate observations made on the temporal lobe from patients with tuberous sclerosis where an accumulation of autophagy proteins such as cargo protein p62, LC3, and ATG12 were found (93). Interestingly, phenotypes observed in iPSC-derived Purkinje cells or neurons from TSC patients were reversed with rapamycin treatment (91, 92).

IPSCs and organoids generated from patients diagnosed with Rett syndrome present with (i) a down-regulation in the expression of genes involved in neuronal development and cell signaling (94) and (ii) decreased expression in genes expressing neuronal markers that include MAP2 and DCX that should reflect deficits in neuronal differentiation (95). Interestingly, inhibition of specific microRNAs 199 and 214 restored MAP2 and DCX expression (Supplementary Table 1).

Two studies that investigated differential gene expression in iPSC-derived neurons from patients diagnosed with idiopathic forms of autism showed a significant downregulation of genes involved in neuronal development and synaptic function (96, 97). A third study focusing on idiopathic autism combined with macrocephaly demonstrated an increased proportion of proliferative cells and a decreased proportion of cells expressing markers of neuronal differentiation (98). Interestingly, differential analyses of whole transcriptomes identified a significant enrichment in ASD-related genes. All these studies demonstrated a decrease in electrophysiological activities (spontaneous activity; calcium transient; or decreased numbers of spikes and bursts) in iPSC-derived neurons from patients affected by ASDs. Application of IGF1 on cultures of iPSC-derived neurons from patients with ASDs led to an increase in the number of spikes (98). A similar treatment was applied to iPSC-derived neurons from a patient carrying a *TRPC6* gene translocation. IGF1 treatment restored dendritic arborization that was decreased in *TPRC6*-disrupted neurons which also had decreased expression in synaptic proteins such as Homer and PSD95 (79). Several phase II clinical trial studies targeting the IGF1 pathway have been performed in Rett, Fragile X, and Phelan McDermid syndromes testing safety and primary efficacy (99).

A screening of 202 compounds was performed on iPSC-derived cortical neurons from patients with *SHANK3* mutations. Two of those molecules, valproic acid (VPA)—an HDAC inhibitor and an antiepileptic drug commonly used for bipolar disease treatment—and lithium, led to an increase in SHANK3 expression and its recruitment to post-synaptic sites (100) as well as increased spontaneous calcium oscillations, improving neuronal network connectivity.

Studies using iPSC-derived cells have shown that common processes are deregulated in syndromic and idiopathic forms of autism characterized by impairment in neuronal differentiation process, an imbalance between inhibitory and excitatory neurotransmission as well as mitochondrial deficits which corroborate genetic data as well as post-mortem brain studies. Several molecules such as rapamycin and IGF1, that have been also tested in mouse models seem to be effective in human iPSC-derived cells. The literature concerning the potential effect of VPA and lithium on mouse models is inconclusive. IPSC-derived cells from patients constitute a critical *in vitro* model for studying the impact of ASD-associated mutations and genetic vulnerability factors on the development and progression of the disease. Thus, many reasons can justify using human iPSC-derived models to increase our understanding of ASDs.

Interestingly, differential expression of human-specific genes was shown between control and brains of patients with ASDs (101). Along these lines, specific families of genes such as transcription factors as well as genes involved in brain size and in the acquisition of language have been submitted to positive selection in the human genome (102, 103). Thus, investigating these genes may not be possible in rodents or non-human primates. Finally, while our current knowledge in epigenetic regulation and non-coding transcriptome is growing (104), it appears that some histone modifications, microRNA or long non-coding RNAs, all of which are potential therapeutic targets to the same extent as proteins, are only observed in human (105, 106).

## Conclusion

The current review aims to bring together findings in the field of ASDs provided by genetics, mouse models, post-mortem brain studies and iPSC-derived studies. All these approaches provide complementary information suggesting that ASDs are underpinned by dysregulation in the brain developmental program which include alterations of activity-dependent development, mitochondrial function as well as an imbalanced excitation-inhibition equilibrium (Figure 1; Supplementary Table 1). Those studies have also highlighted therapeutic strategies that are often primarily performed in mice but remain to be fully translated into humans. However, the human iPSC model seems to be an ideal model system for integrating all the genes and other factors that are implicated as causative factors in the development of ASDs. Furthermore, this review has highlighted the importance of human-specific gene regulation and expression involved in high cognitive and behavioral functions for ASDs. Further investigations involving functional analyses on human models are required to identify molecules and design therapeutic strategies that could be translated to patients.

## Author Contributions

TD, GM, and CR defined the scope of the review. GM and CR wrote the manuscript and prepared the tables. GB, TD, and LB corrected, edited, and formatted the manuscript.

### Conflict of Interest Statement

The authors declare that the research was conducted in the absence of any commercial or financial relationships that could be construed as a potential conflict of interest.
